# Draft Genome Sequence of *Hoeflea* sp. Strain BAL378, a Potential Producer of Bioactive Compounds

**DOI:** 10.1128/genomeA.01213-14

**Published:** 2014-11-20

**Authors:** Mikkel Bentzon-Tilia, Lasse Riemann, Lone Gram

**Affiliations:** a Department of Systems Biology, Technical University of Denmark, Kongens Lyngby, Denmark; b Marine Biological Section, Department of Biology, University of Copenhagen, Helsingør, Denmark

## Abstract

Some phytoplankton-associated marine bacteria produce bioactive compounds. Members of the genus *Hoeflea* may be examples of such bacteria; however, data describing their metabolisms are scarce. Here, we report the draft genome sequence of *Hoeflea* sp. strain BAL378, a putative producer of bacteriocins, polyketides, and auxins, as demonstrated by genome mining.

## GENOME ANNOUNCEMENT

The genus *Hoeflea* belongs to the *Phyllobacteriaceae* family of the *Alphaproteobacteria*. It was established in 2005 (1) as a consequence of the reclassification of the species *Agrobacterium ferrugineum* (a marine, star-shaped, aggregate-forming bacterium) into the novel *Pseudorhodobacter* genus (2). Based on 16S rRNA gene sequence similarity, the *Pseudorhodobacter* genus did not, however, include the strain *A. ferrugineum* LMG 128, which subsequently became the type species of the *Hoeflea* genus, *Hoeflea marina* LMG 128 (1). Currently, the *Hoeflea* genus represents seven species, all of which are isolated from marine and estuarine environments (1, 3–8). One trait commonly shared between the members of *Hoeflea* is the association with primary producers. *Hoeflea suaedae* was originally isolated from a halophyte (6), whereas three other species were isolated from phytoplankton (3–5). In addition to rosette formation, phytoplankton colonization is a trait the *Hoeflea* organisms share with some members of the prevalent marine *Roseobacter* clade. These organisms may facilitate phytoplankton colonization by the production of secondary metabolites that inhibit the growth of potentially competing bacteria (antibiotics) and promote phytoplankton growth (auxins) (9, 10). Whether the similarities between the *Hoeflea* organisms and the *Roseobacter* clade extends to the production of these bioactive compounds is currently unknown, as the *Hoeflea* genus is not yet well described, and only two whole-genome sequences are available (accession numbers ABIA00000000 and ARCZ00000000). Hence, we report here the draft genome sequence of *Hoeflea* sp. BAL378, a strain isolated from Baltic Sea surface water (11).

Genomic DNA was extracted from a culture growing to an optical density at 600 nm (OD_600_) of ≈1 in ZoBell medium (12) using the E.Z.N.A. tissue DNA kit (Omega Bio-Tek, Norcross, GA, USA), and a paired-end sequencing library with inserts of 450 bp was prepared. Indexing was done using short indexing primers (13), and sequencing was done on an Illumina HiSeq 2000 sequencer at the University of Copenhagen sequencing center. The sequencing data represent 6.1 × 10^7^ sequencing reads that were assembled using Velvet *de novo* assembler version 1.2.08, with scaffolding switched off and a k-mer of 47; this produced 282 contigs with a mean length of 19.5 kb, the longest being 239 kb. The unclosed draft genome sequence of *Hoeflea* sp. BAL378 consists of 5.49 Mbp of DNA, with a G+C content of 65.3%. The genome was annotated using the NCBI Prokaryotic Genome Annotation Pipeline and the online Rapid Annotations using Subsystems Technology (RAST) resource version 2.0 (14). Secondary metabolite prediction was done using antiSMASH 2.0 (15) and NaPDoS.

According to antiSMASH, *Hoeflea* sp. BAL378 harbors two bacteriocin gene clusters and two terpene-encoding gene clusters. The RAST annotation suggested that bacteriocins produced by the strain are similar to colicin V and the broad-spectrum antibacterial protein marinocine from the marine bacterium *Marinomonas mediterranea* (16). The annotation also identified four genomic features as being related to enzymes involved in auxin production. Additionally, NaPDoS identified five putative polyketide synthases but no nonribosomal peptide synthases.

### Nucleotide sequence accession numbers.

This whole-genome shotgun project has been deposited at DDBJ/EMBL/GenBank under the accession no. JRJG00000000. The version described in this paper is the first version.
